# Coping strategies related to food insecurity at the household level in Bangladesh

**DOI:** 10.1371/journal.pone.0171411

**Published:** 2017-04-14

**Authors:** Fahmida Dil Farzana, Ahmed Shafiqur Rahman, Sabiha Sultana, Mohammad Jyoti Raihan, Md Ahshanul Haque, Jillian L. Waid, Nuzhat Choudhury, Tahmeed Ahmed

**Affiliations:** 1 Nutrition and Clinical Services Division, International Centre for Diarrhoeal Disease Research, Bangladesh, Dhaka, Bangladesh; 2 James P Grant School of Public Health, BRAC University, Dhaka, Bangladesh; 3 Helen Keller International, Dhaka, Bangladesh; SOAS, University of London, UNITED KINGDOM

## Abstract

**Introduction:**

In connection to food insecurity, adaptation of new techniques or alteration of regular behavior is executed that translates to coping strategies. This paper has used data from food security and nutrition surveillance project (FSNSP), which collects information from a nationally representative sample in Bangladesh on coping behaviors associated with household food insecurity. To complement the current understanding of different coping strategies implemented by the Bangladeshi households, the objective of this paper has been set to examine the demographic and socio-economic characteristics of the food insecure households which define their propensity towards adaptation of different types of coping strategies.

**Methodology:**

FSNSP follows a repeated cross-sectional survey design. Information of 23,374 food insecure households available from February 2011 to November 2013 was selected for the analyses. Coping strategies were categorized as financial, food compromised and both. Multinomial logistic regression was employed to draw inference.

**Results:**

Majority of the households were significantly more inclined to adopt both multiple financial and food compromisation coping strategies. Post-*aman* season, educational status of the household head and household women, occupation of the household’s main earner, household income, food insecurity status, asset, size and possession of agricultural land were found to be independently and significantly associated with adaptation of both financial and food compromisation coping strategies relative to only financial coping strategies. The relative risk ratio of adopting food compromisation coping relative to financial coping strategies when compared to mildly food insecure households, was 4.54 times higher for households with moderate food insecurity but 0.3 times lower when the households were severely food insecure. Whereas, households were 8.04 times and 4.98 times more likely to adopt both food compromisation and financial relative to only financial coping strategies if moderately and severely food insecure respectively when compared to being mildly food insecure.

**Conclusion:**

Households suffering from moderate and severe food insecurity, are more likely to adopt both financial and food compromisation coping strategies.

## Introduction

Food security is a complex sustainable development issue linked to health and nutrition, has been best defined by the World Food Summit as having access to sufficient, safe and nutritious food [1]. Food insecurity, the converse situation can be described as "limited or uncertain availability of nutritionally adequate and safe foods or limited or uncertain ability to acquire acceptable foods in socially acceptable ways" [2]. Food insecurity indeed is a major public health problem for both developing and developed nations [3]. Historically, household resilience to food insecurity has been characterized by a number of fairly regular behavioral responses which translates to coping strategies [4] or techniques that households generally apply to cope with crises moments when the resources are limited or absent [5].Generally, households adopt coping strategies in the early stages of food insecurity [6], which however vary based on cultural and geographical differences [5].

Food insecure households reportedly exhibit a range of coping techniques that reflects their vulnerability [6]. In the phase of idiosyncratic shocks such as food price hike or natural disasters, households may employ food or non-food based coping strategy or a combination of both to protect their basic needs [7,8]. In recently conducted studies, several coping strategies were found to be associated with household food insecurity, food consumption at household and individual level. Poverty measures as income and expenditure and seasonal variation of staple food production are also related to coping strategies [9–14]. Previous experiences have indicated that, during idiosyncratic shocks such as food price hike, poor households adopt a series of coping strategies which can be differentiated as food and non-food based techniques. Purchasing less preferred food, reducing meal size, consuming only rice, skipping meals and selling of assets were the frequently reported responses at the time of food shortage [4,15–19]. These coping strategies were also commonly observed in the context of Bangladesh, a densely populated lower-middle income country which often encounters natural calamities resulting in around 40% of its households being food insecure [20,21].

Literatures have identified diverse coping strategies applied at the household level amongst population affected by natural calamity and food price shock, but not in a general population who also tend to cope at a regular basis due to food insecurity at the household level. Particularly the contexts that compel households to apply only food compromised or financial coping strategies, are not well defined. This paper is based on data collected through the food security and nutrition surveillance project (FSNSP), the single surveillance system in Bangladesh to look upon the coping behaviors of food insecure households countrywide [22]. Understanding the implemented coping strategies at household level is critical for formulating and implementing appropriate policy and design programs related to food insecurity. The objective of this paper has been set to examine the relationship of different categories of food insecurity with types of coping strategies. This is expected to complement the current understanding of different coping strategies pertaining to food insecurity implemented by the Bangladeshi households. Moreover, this paper also tried to identify the significant demographic and socio-economic characteristics of the food insecure households that define their propensity towards adaptation of these strategies.

## Methodology

FSNSP covers three major seasons in Bangladesh: monsoon (May-August) and the two post rice harvest periods namely post-*aman*(January-April) and post-*aus* (September-December).FSNSP collects information on food insecurity at the household level from 13 strata; six strata correspond to the six surveillance zones(coastal belt, eastern hills, haor region, padma chars, northern chars and the northwest region), while the remaining seven strata(Dhaka, Chittagong, Rajshahi, Barisal, Khulna, Sylhet and Rangpur), which contain all the upazila not included in a surveillance zone, correspond to the seven administrative divisions of Bangladesh. From each stratum, a set number of upazila were selected with replacement. For each of the six surveillance zones, twelve upazila were selected in each round, while 22 upazila were selected from the other areas of the country. The number of upazila from non-surveillance zone strata varied depending on the number of upazila in the zone, ranging from one to eight.1 From each of the surveillance zones, upazila were selected by rotation into the sampling frame in order to reduce random variation in estimates between rounds, as has been recommended for surveillance systems by the UN (United Nations), and is commonly done in labour participation surveillance [23].

### Study design and sample size

**Fig 1:**
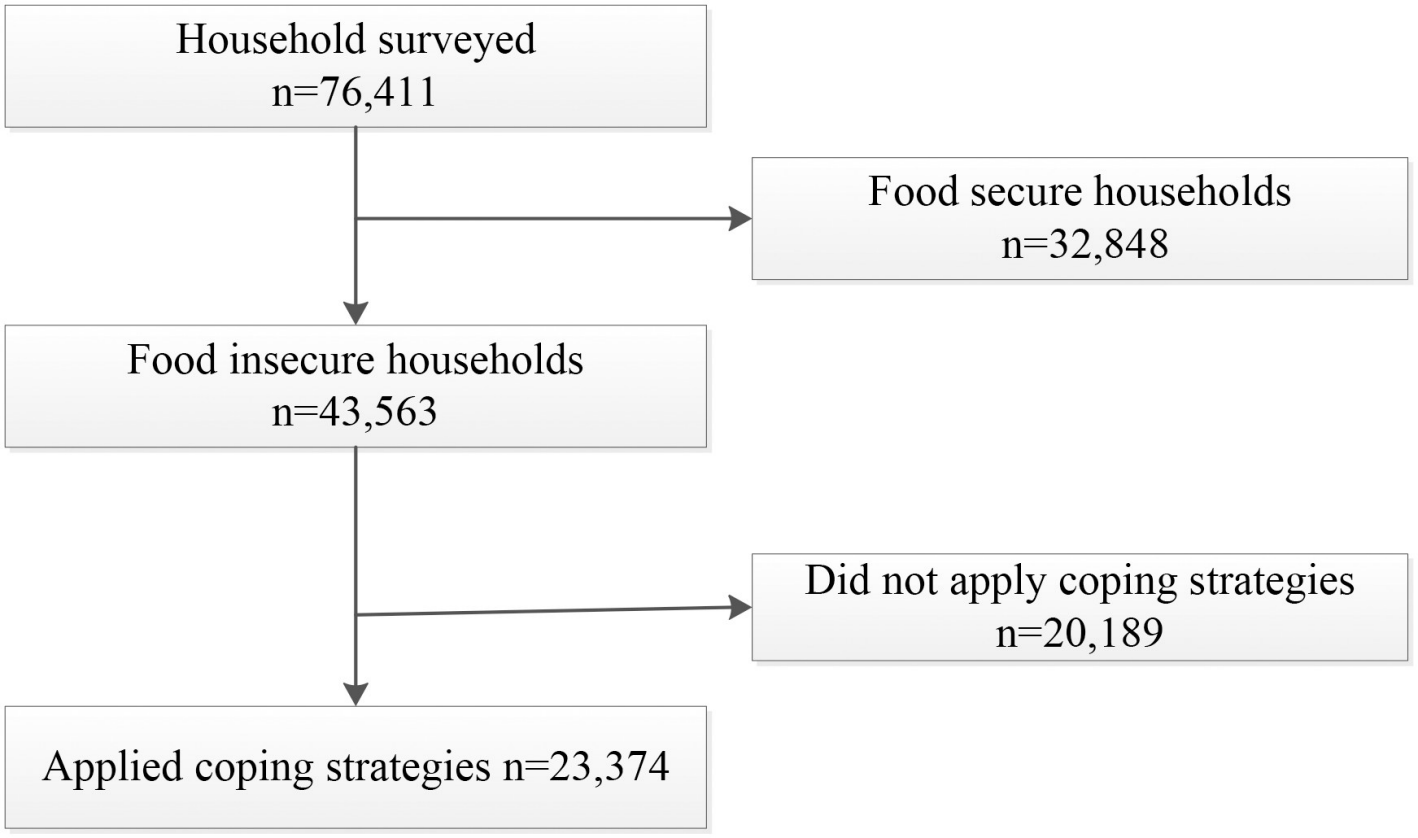
Study profile.

For the surveillance, FSNSP followed a repeated cross-sectional survey and data collection occurred every four months [22].The target sample size for FSNSP surveillance was calculated considering prevalence of child wasting, underweight and stunting, women’s chronic energy deficiency and household food insecurity status. Formula for a single population proportion with 95% confidence interval and 5% precision was involved for calculating sample size. Details of sample size estimation and method could be found in the FSNSP annual reports [22]. A total of 23,374 food insecure households available from February 2011 to November 2013 (9 data collection points within 3 years) who applied coping strategies were chosen for the current analyses (Fig 1).

### Data collection and analysis

Data was collected through structured interview facilitated by paper based questionnaire and personal digital assistants (PDAs) both. In each round, 36 two-member teams were involved for collection of data. Quality control was done in around 10% randomly chosen cases within 24 hours of data collection. STATA (version 10) was employed for conducting the analysis. The analysis primarily involved descriptive statistics using appropriate cut-off values for particular variables. Multinomial logistic regression was used to establish both crude and confounder adjusted relationship between the outcome and response variables. In the multinomial logistic regression models, Relative Risk Ratio (RRR) with 95% CI was noted and variables were considered as significant predictors if the p-value was less than 0.05.

### Variables of interest

**Fig 2:**
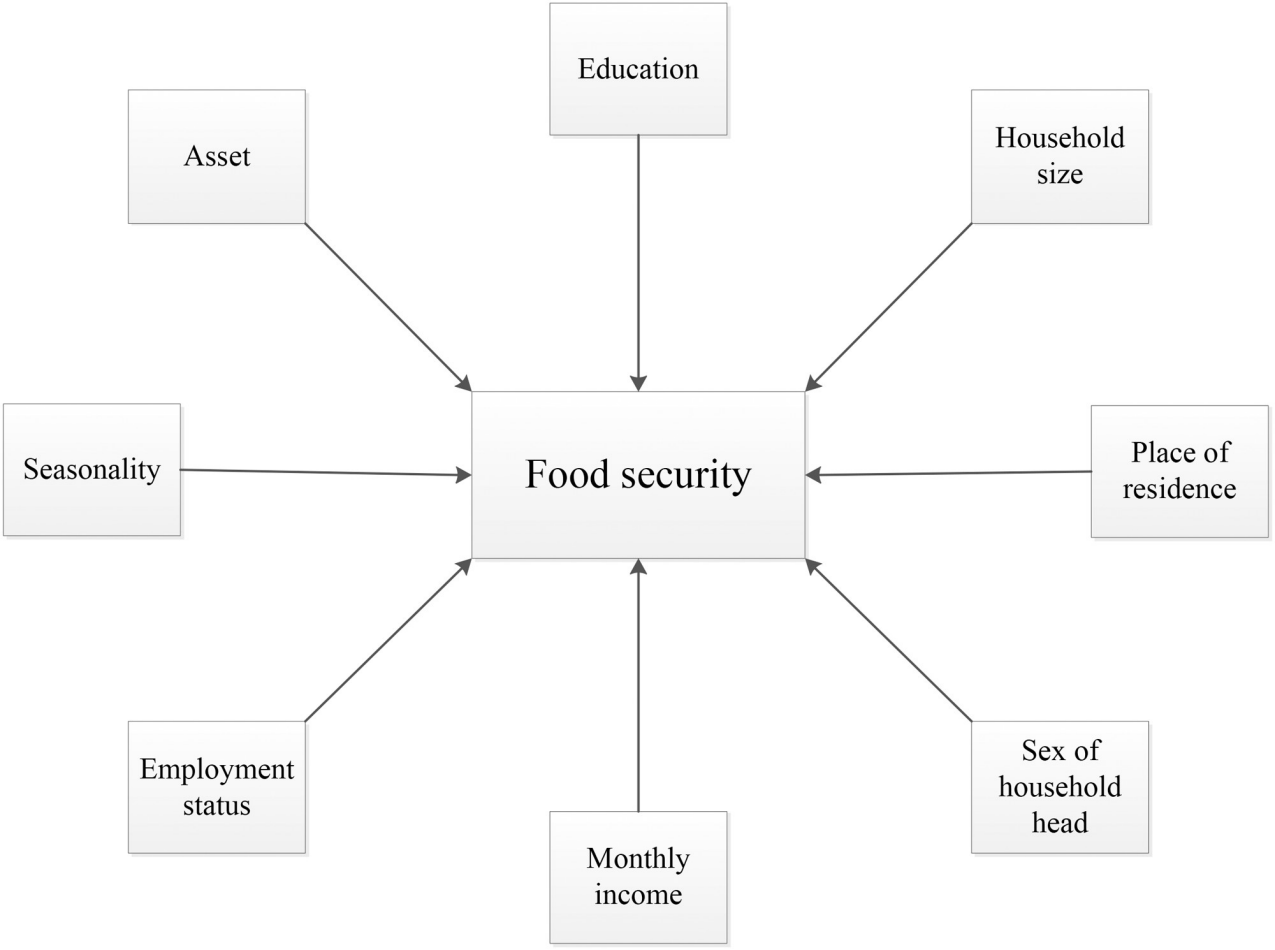
Factors influencing household food security status.

Data of previous month (30 days) was collected on food insecurity at the household level using questions to understand the level of access to food and was categorized as mild, moderate and severe according to their score at Household Food Insecurity Access Scale (HFIAS) [24]. HFIAS determines food insecurity based on lack of access due to poverty rather than shortage of supply [25].The scale is comprised of 9 questions (worry about food, unable to eat preferred foods, eat just a few kinds of foods, eat foods they really do not want eat, eat a smaller meal, eat fewer meals in a day, no food of any kind in the household, go to sleep hungry, go a whole day and night without eating) to assess the level of anxiety and uncertainty of the participants about household food supply, insufficient quality of food and insufficient food intake [24].The six coping strategies adopted by the food insecure households were namely sale or mortgage of assets, consumption of low quality food, consumption of fewer items of food, stop schooling of household members, borrowing food and borrowing money. The outcome variable was categorized into only financial coping (sale or mortgage of assets, stop schooling of household members, borrowing money and food), only food compromisation coping (consumption of low quality food and consumption of fewer items of food) and both financial and food compromisation coping strategies. Since the dependent/outcome variable had more than two categories, multinomial logistic regression was used with “financial coping” as the base outcome. Considering the relevant predictors of household food insecurity as found in relevant papers during our literature review, thirteen variables were considered for subsequent multivariate analysis (Fig 2). The selected response variables were seasonality [4,26], residence type [7,27], sex of the household head [28–31], education level of the household head [3,28,32,33], occupation of primary earner [9,34], agricultural land of the household [35,36], household homestead gardening [37], household monthly income [3,38,39], education [9,31,38,40–42] and occupational status [9] of the households’ women, household food insecurity status, asset index [41,43,44], and number of household members [45,46]. Asset index was constructed through principal component analysis as used in Bangladesh Demographic and Health Survey [47].

### Ethical consideration and consent procedure

This study was approved by the research review committee and ethical review committee, the two obligatory components of the institutional review board of International Centre for Diarrhoeal Disease Research, Bangladesh (icddr,b). Verbal informed consent was taken instead of written due to the cultural stigma towards signing paper documents especially in rural areas of Bangladesh. At the beginning of each interview, the data collection officers informed the respondent about the purpose of surveillance through reading a consent statement aloud. They were also informed about their participation to be voluntary and it is also allowed to withdraw their participation at any time. Verbal consent from the caretaker was also taken regarding anthropometric measurement of the study child.

## Results

Descriptive statistics derived from the analysis, are tabulated in Table 1.Our result dictates that around four-fifth of all food insecure households were severely food insecure, mostly belonged to rural areas, majority of the families were headed by male members. As for the household heads, around half had no formal education and major occupation was day labor.

**Table 1:** Descriptive statistics of food insecure households applying coping strategies.

**Continuous variable**		**Mean (95% CI)**
Household size		4.81 (4.80–4.84)
**Categorical variables**		**n (%)**
Household food insecurity	Mildly food insecure	1901 (8.13)
	Moderately food insecure	1977 (8.46)
	Severely food insecure	19496 (83.41)
Residential area	Rural	21506 (92.01)
	Urban	1868 (7.99)
Seasonality	Post-aus	7362 (31.5)
	Post-aman	8164 (34.93)
	Monsoon	7848 (33.58)
Sex of household head	Male	20989 (89.8)
	Female	2385 (10.2)
Education level of household head	SSC complete and above	1193 (5.11)
	Below SSC	9770 (41.88)
	No formal education	12363 (53)
Occupation of household head	Farmer	4611 (19.73)
	Day laborer	11815 (50.55)
	Businessman	2999 (12.83)
	Professional wage earner	1535 (6.57)
	No income	2216 (9.48)
	Others	198 (0.85)
Occupation of primary earner	Farmer	4283 (18.32)
	Businessman	3121 (13.35)
	Day labor	12911 (55.24)
	Professional wage earner	2112 (9.04)
	Foreign employment	722 (3.09)
	No income	46 (0.2)
	Others	179 (0.77)
At least one women with income generating activity in the household		9044 (38.69)
At least one educated women in household		19624 (83.96)
Possession of agricultural land		6144 (26.29)
Possession of homestead gardening		14385 (61.54)
Beneficiary of at least one safety net program		9151 (39.15)
Received remittance from abroad		2416 (10.34)
Household income (last month) (Tk.)	<3000	6199 (26.52)
	3000 to <6000	8000 (34.23)
	6000 to <10000	5239 (22.41)
	10000 to <20000	2457 (10.51)
	≥ 20,000	1479 (6.33)
Asset index*	1^st^ quintile	5738 (24.55)
	2nd quintile	5830 (24.94)
	3rd quintile	5054 (21.62)
	4th quintile	3600 (15.4)
	5th quintile	3152 (13.49)

*5^th^ quintile = richest, 4^th^ quintile = richer, 3^rd^ quintile = middle, 2^nd^ = poorer, 1^st^ = poorest

**Fig 3:**
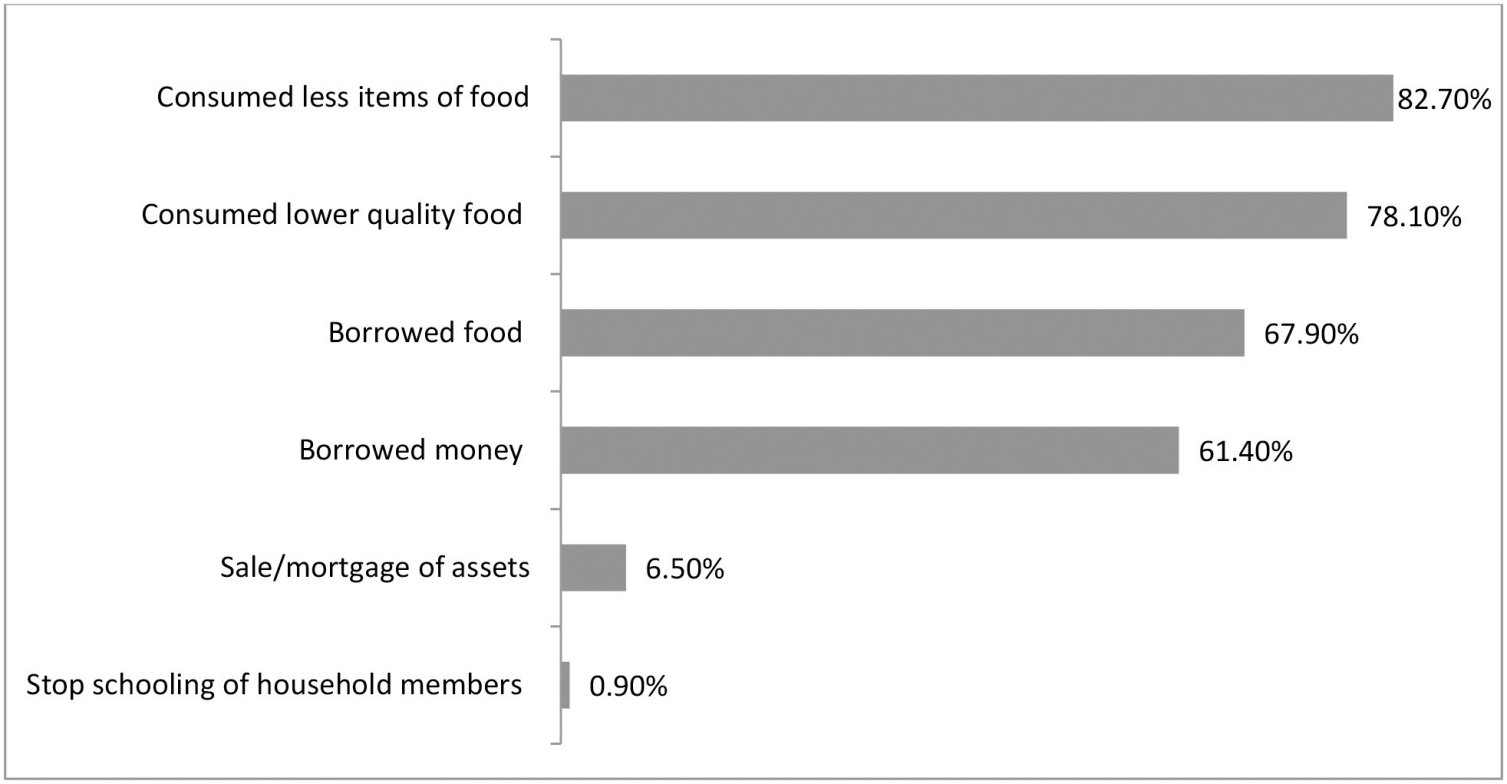
Adopted coping strategies of the household [multiple response].

**Fig 4:**
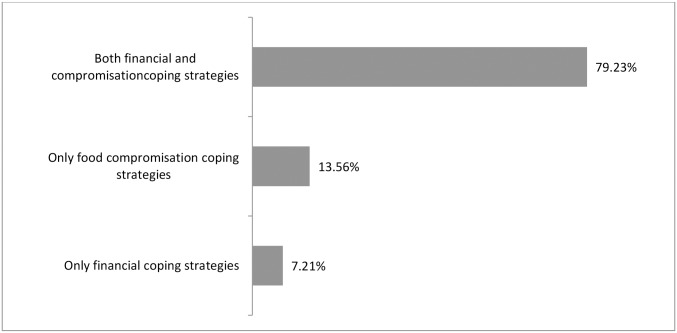
Categories of coping strategies adopted by household.

**Fig 5:**
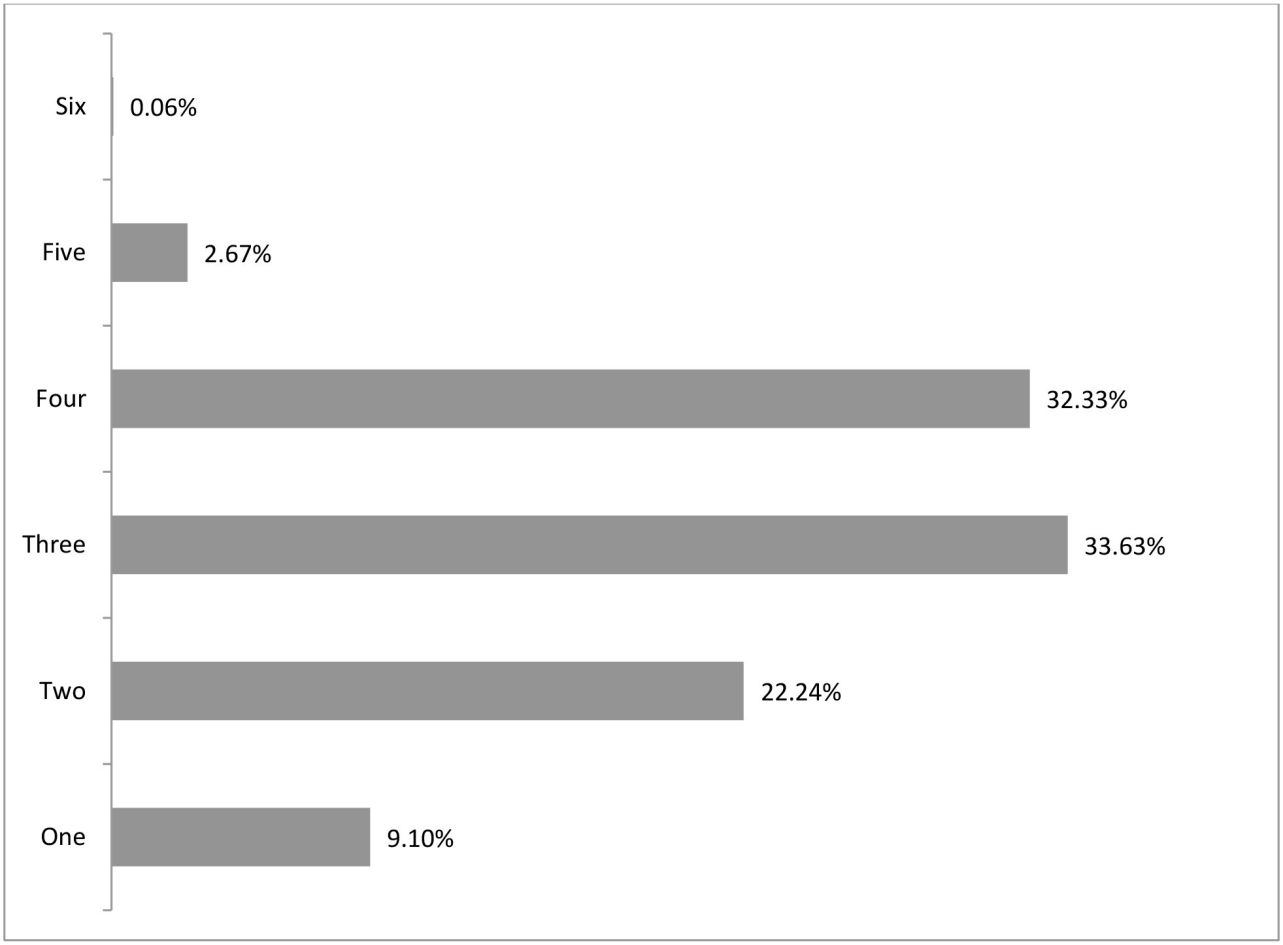
Number of coping strategies adopted by the households.

**Fig 6:**
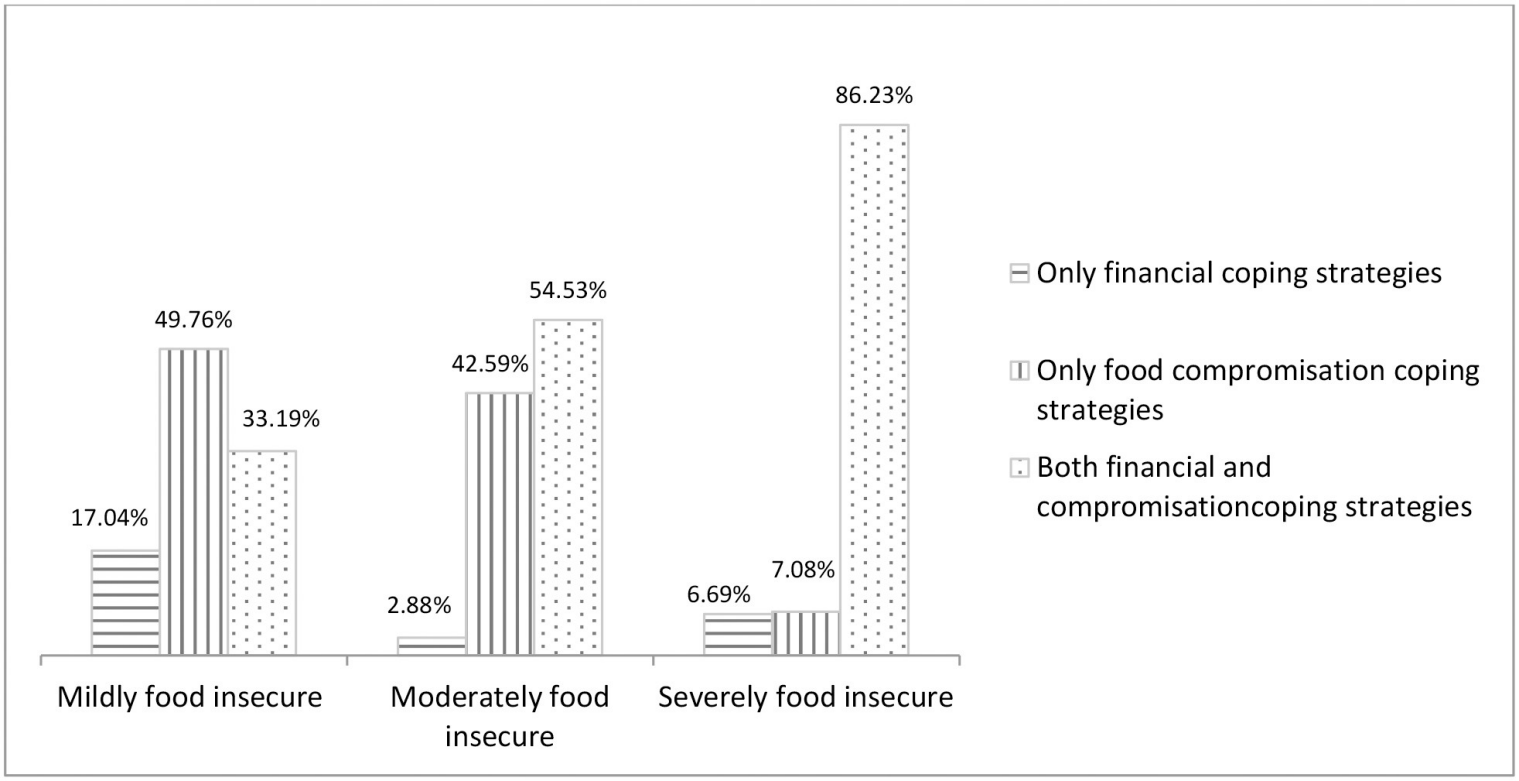
Categories of coping strategies by household stratefied by food insecurity status.

Compromising the quality and quantity of food were the two most common coping strategies adopted (Fig 3) and when the coping strategies were categorized, 79.2% households adopted both financial and food compromising strategies (Fig 4) in general. One third of the households applied three to four coping strategies (Fig 5). Moreover, more than 85% of severely food insecure households (Fig 6) implemented mixed compromisation strategies of both financial and food domain.

Table 2 represents the determinants of food compromised coping strategies and both food compromised and financial coping strategies while the variables were unadjusted.

**Table 2:** Determinants (unadjusted) of coping strategies at the household level (Outcome: Food compromised coping and both food compromised and financial coping; reference category: Financial coping).

Variables		n	Unadjusted RRR (95%CI) of food compromised coping	p-value	Unadjusted RRR (95%CI) of both food compromised and financial coping	p-value
Seasonality		23374				
	Post-aus		Reference		Reference	
	Post-aman		0.55 (0.41–0.74)	<0.001	0.64 (0.51–0.8)	<0.001
	Monsoon		0.7 (0.53–0.94)	0.018	0.77 (0.61–0.98)	0.031
Residential area		23374				
	Urban		Reference		Reference	
	Rural		0.99 (0.69–1.41)	0.947	1.3 (0.97–1.76)	0.083
Sex of household head		23374				
	Male		Reference		Reference	
	Female		0.92 (0.74–1.14)	0.449	0.86 (0.72–1.03)	0.094
Education level of household head		23326				
	SSC complete and above		Reference		Reference	
	Below SSC		1.21 (0.97–1.51)	0.094	1.83 (1.54–2.19)	<0.001
	No formal education		1.58 (1.25–2)	<0.001	2.82 (2.33–3.41)	<0.001
Occupation of primary earner		23374				
	Farmer		Reference		Reference	
	Businessman		1.3 (1.09–1.56)	0.005	2.11 (1.81–2.47)	<0.001
	Day labor		1.28 (1.03–1.58)	0.026	1.18 (0.98–1.42)	0.086
	Professional wage earner		0.99 (0.79–1.24)	0.931	0.93 (0.76–1.14)	0.509
	Foreign employment		0.86 (0.6–1.23)	0.399	0.73 (0.56–0.96)	0.027
	Others		0.81 (0.37–1.75)	0.587	0.94 (0.52–1.7)	0.835
	No income		1.23 (0.21–7.32)	0.823	2.64 (0.59–11.79)	0.202
Agricultural land		23374				
	Some agricultural land		Reference		Reference	
	No agricultural land		1.16 (0.99–1.34)	0.059	1.86 (1.65–2.1)	<0.001
Homestead gardening		23374				
	Yes		Reference		Reference	
	No		1.11 (0.96–1.28)	0.148	1.18 (1.05–1.33)	0.007
Income of last month (Tk)		23374				
	Above 20,000		Reference		Reference	
	10000 to <20000		1.21 (0.93–1.57)	0.162	1.28 (1.01–1.62)	0.045
	6000 to <10000		1.46 (1.14–1.86)	0.003	2.05 (1.66–2.52)	<0.001
	3000 to <6000		1.54 (1.21–1.96)	<0.001	2.93 (2.37–3.63)	<0.001
	Below 3000		1.62 (1.24–2.11)	<0.001	3.47 (2.76–4.36)	<0.001
Women education status		23374				
	At least 1 women with some education		Reference		Reference	
	No women with any formal education		1.64 (1.32–2.03)	<0.001	1.65 (1.37–1.98)	<0.001
Women with income generating activity (IGA)		23374				
	At least 1 women with IGA		Reference		Reference	
	No IGA		0.96 (0.83–1.11)	0.593	0.8 (0.71–0.9)	<0.001
Household food insecurity		23374				
	Mildly food insecure		Reference		Reference	
	Moderately food insecure		5.06 (3.67–6.98)	<0.001	9.71 (6.94–13.59)	<0.001
	Severely food insecure		0.36 (0.29–0.45)	<0.001	6.62 (5.5–7.97)	<0.001
Asset index*		23374				
	5^th^ quintile		Reference		Reference	
	4^th^quintile		1.01 (0.83–1.22)	0.951	1.19 (1–1.41)	0.051
	3^rd^quintile		1.05 (0.85–1.31)	0.654	1.62 (1.36–1.93)	<0.001
	2^nd^quintile		1.17 (0.92–1.48)	0.204	2.13 (1.76–2.58)	<0.001
	1^st^quintile		1.21 (0.95–1.54)	0.130	2.12 (1.73–2.59)	<0.001
Household size		23374	1.02 (0.98–1.07)	0.285	1.06 (1.02–1.1)	0.002

*5^th^ quintile = richest, 4^th^ quintile = richer, 3^rd^ quintile = middle, 2^nd^ = poorer, 1^st^ = poorest

Table 3 illustrates the determinants of only food compromised coping and both food compromised and financial coping when adjusted. Our result also indicates the existence of significant association between different types of coping strategies and the level of existing household food insecurity. The result implies that compared to mildly food insecure, severely food insecure households were significantly more inclined to adopt both financial and food compromisation coping strategies. Whereas, moderately food insecure households were also found to be significantly more opted to implement mixed coping strategies rather than only financial coping strategies. However, moderate food insecure households were significantly more likely to adopt food compromisation coping relative to only financial coping strategies but for severely food insecure households, that RRR were significantly less.

**Table 3:** Determinants (adjusted) of coping strategies at the household level (Outcome: Food compromised coping and both food compromised and financial coping; reference category: Financial coping)*.

Variables		n	Adjusted RRR (95%CI)* of food compromised coping	p-value	Adjusted RRR (95%CI) of both food compromised and financial coping	p-value
Seasonality		23374				
	Post-aus		Reference		Reference	
	Post-aman		0.6 (0.44–0.82)	0.001	0.71 (0.57–0.89)	0.003
	Monsoon		0.75 (0.56–1.01)	0.060	0.87 (0.69–1.1)	0.259
Residential area		23374				
	Urban		Reference		Reference	
	Rural		0.84 (0.57–1.25)	0.394	1.14 (0.86–1.53)	0.363
Sex of household head		23374				
	Male		Reference		Reference	
	Female		1.13 (0.89–1.43)	0.320	1.01 (0.82–1.23)	0.950
Education level of household head		23326				
	SSC complete and above		Reference		Reference	
	Below SSC		1.1 (0.86–1.39)	0.452	1.17 (0.96–1.44)	0.119
	No formal education		1.31 (1.01–1.71)	0.043	1.37 (1.1–1.71)	0.005
Occupation of primary earner		23374				
	Farmer		Reference		Reference	
	Businessman		1.21 (0.99–1.47)	0.064	1.8 (1.52–2.14)	<0.001
	Day labor		1.23 (0.98–1.54)	0.072	1.41 (1.16–1.71)	<0.001
	Professional wage earner		1.08 (0.84–1.38)	0.550	1.26 (1.02–1.57)	0.032
	Foreign employment		1.09 (0.71–1.69)	0.696	1.54 (1.1–2.15)	0.012
	Others		0.85 (0.4–1.79)	0.660	1.42 (0.82–2.48)	0.212
	No income		1.01 (0.16–6.4)	0.988	2.72 (0.61–12.25)	0.191
Agricultural land		23374				
	Some agricultural land		Reference		Reference	
	No agricultural land		1.12 (0.95–1.33)	0.174	1.37 (1.19–1.58)	<0.001
Homestead gardening		23374				
	Yes		Reference		Reference	
	No		1.2 (1.04–1.4)	0.016	1.1 (0.97–1.25)	0.141
Income of last month (Tk)		23374				
	Above 20,000		Reference		Reference	
	10000 to <20000		1.13 (0.84–1.52)	0.429	1.03 (0.8–1.33)	0.838
	6000 to <10000		1.38 (1.05–1.82)	0.021	1.42 (1.12–1.8)	0.003
	3000 to <6000		1.61 (1.23–2.12)	0.001	1.92 (1.51–2.45)	<0.001
	Below 3000		1.72 (1.29–2.29)	<0.001	2.58 (2.01–3.32)	<0.001
Women education status		23374				
	At least 1 women with some education		Reference		Reference	
	No women with any formal education		1.63 (1.3–2.04)	<0.001	1.32 (1.09–1.61)	0.004
Women with income generating activity (IGA)		23374				
	At least 1 women with IGA		Reference		Reference	
	No IGA		0.89 (0.76–1.04)	0.131	0.85 (0.74–0.96)	0.010
Household food insecurity		23374				
	Mildly food insecure		Reference		Reference	
	Moderately food insecure		4.54 (3.3–6.25)	<0.001	8.04 (5.75–11.26)	<0.001
	Severely food insecure		0.3 (0.25–0.38)	<0.001	4.98 (4.13–6.01)	<0.001
Asset index**		23374				
	5^th^ quintile		Reference		Reference	
	4^th^quintile		0.95 (0.78–1.16)	0.614	1.02 (0.85–1.22)	0.838
	3^rd^quintile		1 (0.8–1.25)	0.997	1.14 (0.95–1.37)	0.151
	2^nd^quintile		1.11 (0.87–1.42)	0.396	1.34 (1.11–1.62)	0.003
	1^st^quintile		1.2 (0.93–1.54)	0.159	1.32 (1.07–1.62)	0.009
Household size		23374	1.07 (1.02–1.12)	0.006	1.14 (1.1–1.19)	<0.001

*Sex of the household head, residence, and seasonality were adjusted in the model;

**5^th^ quintile = richest, 4^th^ quintile = richer, 3^rd^ quintile = middle, 2^nd^ = poorer, 1^st^ = poorest

While on other socio-demographic determinants of food compromisation relative to financial coping strategies, our result indicates educational level of household head, occupation of primary earner, absence of homestead gardening, household income level, women education status and household size as significant predictors. Whereas, educational level of household head, occupation of primary earner, possession of agricultural land and homestead gardening, household income, household women education status, asset index and household size were significant predictors for adaptation of both financial and food compromisation in comparison to only financial coping strategies.

## Discussion

Coping strategies pertaining to compromising quality and quantity of food consumption were observed to be the first step taken in order to mitigate the adverse effect of food shortage at the household level [18].More exorbitant strategies involving financial compromisation such as selling or mortgaging assets were adopted when food insecurity condition worsens. Literature on the topic is relatively scarce and lack inference based on quantitative analysis. Nonetheless, a study conducted on Bangladeshi marginal farmers affected by idiosyncratic shocks showed compromising the frequency and amount of food to be the most common coping strategy implemented by the households followed by consumption of wild uncultivated food and taking loans [20]. The study also found that as assisted coping strategy, over two-third of the population opted for food relief provided through different safety net programs by the government, non-governmental organizations (NGOs) or other organizations. Another study on the economically vulnerable *haor* zone of Bangladesh showed that nearly 80% of the households primarily preferred borrowing money to circumvent poverty and food insecurity, while half of the population also implemented food compromisation strategies [48]. This study result, in concordance with our finding, also showed that the coping strategies adopted by the vulnerable households were not mutually exclusive, rather a mixed approach comprising strategies of multiple financial and food compromisation domain were adopted. Prior work on household food insecurity suggested that families access an array of informal assistance programs and that they also use financial coping mechanisms i.e. selling assets; these informal assistances are the social safety-net programs can help alleviate food insecurity [19]. However, it is crucial to highlight that in Bangladesh, safety net programs run by government aim to mitigate food insecurity, involves transfer of food mostly [49]. The top few social safety net programs in Bangladesh are the Vulnerable Group Development (VGD) [50,51] with more than 480,000 recipient households [52], the Food for Work (FFW) [52,53] serving more than 75,000,000 hours of work and the Vulnerable Group Feeding (VGF) [54], which are all food oriented. Therefore, considering the inclination of the moderate and severe food insecure households towards adaptation of mixed food and financial compromisation strategies, it should be highly advisable that the government and the NGOs modify their existing food insecurity alleviation oriented safety net programs and incorporate financial modalities such as cash/asset transfer or small loans alongside with food transfer. Comparison of food and cash transfer programs in Bangladesh has shown increased caloric intakes of school age children and elderlies if they are benefited by cash transfer programs [55], however, irregularity in receiving cash payments in terms of timeliness has challenged its efficacy [56].

On the seasonal dynamics of adopted coping strategies, is it needed to be mentioned that rice is the staple cereal grain and the fundamental driver of the agro-based economy of Bangladesh [43,57]. However, rice production is invariably related to seasonal variation and the interim period between different harvests threatens the employment opportunities of around 75% of the population who depends on the agricultural sector as the primary means of livelihood [58,59]. Food insecurity prevails during the transitory post-harvest periods [43] due to seasonal unemployment and lack of food stock which forces households to adopt different coping strategies. In Bangladesh, the *post-aus* season between September to December observes less severity of household food insecurity due to the boosted cumulative harvest of two varieties of rice in the time period [60], coupled with employment availability for the upcoming winter crop transplantation [61,62]. Our result suggests that despite having no difference between post-*aman* and *monsoon* season, households were less likely to adopt both food compromisation and financial coping strategies during the post*-aus* season. It is noteworthy that, when coping strategies are originated following a crisis, they can also lead to a new livelihood pattern [11] which this study could not illustrate.

Our study has illustrated that, moderate food insecure households were significantly more likely to adopt food compromisation coping relative to only financial coping strategies but for severely food insecure households, significantly less RRR was observed. This finding highlights the reduced tendency of severely food insecure households towards food compromisation strategies comparing to financial coping strategies which is perhaps due to the pragmatic scenario that severely food insecure households never have enough food at reserve which could be further compromised. While on other socio-demographic determinants of food compromisation and both food compromisation and financial coping relative financial coping strategies, our result indicated education level of household head, household income level, household size and women education status as significant predictors. Our findings of the significant association between coping strategy and education level of household head supports similar findings from previously conducted studies [9,45,63]. In concordance with our study, household income level was also reported by other studies to be significantly associated with food insecurity derived coping strategies [63,64]. Households with large family size are food insecure compared to those with small numbers of members, which favors previous study findings [45,46]. We have also found women education status as a significant determinant of coping strategy previously reported [9]. Educated women may have their established role or voice in household decision making, which in turn could influence household food insecurity situation as well as adaptation of coping strategies.

## Limitations and strengths

The study did not look upon the causes behind the households being food insecure; i.e. the situation that compelled them to apply different coping strategy and whether they got back to a normal situation thereafter. Data was derived through cross sectional surveillance from which, causal relationships cannot be determined. A possibility of recall bias remains, as information was gathered mostly through maternal response. Nevertheless, a large sample size added to the strength of the study.

## Conclusion

This study is the first of its kind to examine the relationship between the degrees of severity of household food insecurity and the types of coping strategies adopted by Bangladeshi households. The study showed that, majority of the households were significantly more inclined to adopt both financial and food compromisation coping strategies. Moreover, severe and moderately food insecure households were more likely to adopt both food compromisation and financial coping strategies when compared to being mildly food insecure. Adopting coping strategies decrease the vulnerability of the poor, exacerbating the scope for breaking the cycle of poverty. Support for further analysis and deeper understanding of people’s livelihood and coping mechanisms in order to strengthen their livelihood and enhance the effectiveness of assistance programs is advisable. The evidence gathered and subsequently shown in this paper along with the recommendation is expected to be vital for the policymakers and NGO personnel to formulate and instrumentalize in new interventions in the existing safety net programs.

## Supporting information

S1 Data Description: Data file (in Stata). (DTA)
